# Phase transition and phase separation in multiferroic orthorhombic Dy_1−x_Ho_x_MnO_3_ (0 ≤ *x* ≤ 1)

**DOI:** 10.1038/srep06506

**Published:** 2014-09-30

**Authors:** Na Zhang, Shuai Dong, Zhaoming Fu, Zhibo Yan, Fanggao Chang, Junming Liu

**Affiliations:** 1 Henan Province Key Laboratory of Photovoltaic Materials, Henan Normal University, Xinxiang 453007, China; 2 Laboratory of Solid State Microstructures, Nanjing University, Nanjing 210093, China; 3 Department of Physics, Southeast University, Nanjing 211189, China

## Abstract

We report on structural, magnetic, ferroelectric, and thermodynamic properties of polycrystalline orthorhombic manganites Dy_1−x_Ho_x_MnO_3_ for Ho substitution levels 0 ≤ *x* ≤ 1. This system offers a possibility to systemically modulate the multiferroicity of *R*MnO_3_ via tuning the *A*-site ionic radii as well as the *A*-site magnetism. The successive transition of the multiferroic ground state is traced from the *bc*-cycloidal (DyMnO_3_) to the *E*-type antiferromagnetic phase (HoMnO_3_). In the middle substitution range 0.4 < *x* < 0.5, the phase separation is prominent, which's residual may survive in an even wider range. Accompanied with the phase transition and phase separation, obvious enhancement of both the polarization and magnetoelectric response is observed. Our experimental study also confirmed that the rare earth (Dy/Ho)-Mn exchange striction is a crucial role in deciding the multiferroicity of manganites.

Perovskite multiferroic manganite *R*MnO_3_ (*R* is a small size trivalent rare-earth ion), with its ferroelectricity originated from special magnetic structures, is one unique platform to study the so-called type-II multiferroics^1^,^2^,^3^,^4^,^5^,^6^. The intrinsic mutual coupling between magnetism and ferroelectricity in these compounds is of great interest to not only the fundamental physics but also promising applications^7^,^8^,^9^,^10^,^11^,^12^. According to the origin of ferroelectric polarization (*P*), perovskite multiferroic *R*MnO_3_ can be classified into two types: the first one relied on the cycloidal Mn-spin structure, with the inverse Dzyaloshinskii-Moriya interaction being the driving force, as represented by TbMnO_3_ and DyMnO_3_ (DMO)^7^,^8^,^10^,^11^,^12^,^13^,^14^,^15^,^16^,^17^. The second one is driven by the symmetric exchange striction, and needs the up-up-down-down or *E*-type antiferromagnetic (*E*-AFM) spin order^18^,^19^,^20^, as defined by orthorhombic HoMnO_3_ (HMO) and YMnO_3_ (YMO). The latter mechanism was predicted to generate much a larger ferroelectric polarization than the first one^18^,^19^,^20^.

Referring to the well-known phase diagram of multiferroic orthorhombic *R*MnO_3_^7^,^8^, the cycloidal order in DMO is proximate to the *E*-AFM order in HMO. The only difference is due to the tiny difference of *A*-site ionic radius: Dy^3+^ (91.2 pm) and Ho^3+^ (90.1 pm)^21^. However, such a tiny difference drives the contrastive spin structures of *R*MnO_3_ changing from the spiral-spin-order (SSO) to the *E*-AFM structure, separated by a first-order phase transition boundary^22^,^23^,^24^. The different spin structure finally leads to the different multiferroic mechanisms^18^,^19^,^25^.

Recently, both theoretical and experimental results revealed that the symmetric (***S***_i_**·*S***_j_)-type magnetostriction also has a significant contribution to the ferroelectricity in the spin-spiral-based multiferroic phase, while the antisymmetric (***S****_i_* × ***S****_j_*)-type magnetostriction may also plays an important role in deciding the ferroelectricity of the *E*-type spin structure^26^,^27^,^28^,^29^,^30^. Specifically, the rare-earth magnetism, which was often ignored in aforementioned two mechanisms, has been argued to be non-negligible for the multiferroicity of both DMO and HMO^26^,^27^,^28^,^29^,^30^. In this sense, the *A*-site rare-earth element not only determine the crystal structure but also directly affect the multiferroicity of *R*MnO_3_. This feature reminds us that one can control the whole spin structure (not only Mn's spin structure) and then tune the multiferrociticy of *R*MnO_3_ by modulating the *A*-site ions.

In addition, due to the mutual coupling between the charge, spin, orbital, and lattice degrees of freedom in manganites, fascinating physical phenomena always emerge during the phase transition process, especially at phase boundaries. For undoped manganites, there are two main phase boundaries in the phase diagram: 1) the A-type antiferromagnetic (*A*-AFM) phase to SSO phase; and 2) the SSO phase to *E*-AFM phase. Thus, it is expected that multiferroic phase separation and possible gigantic magnetoelectric (ME) effect will turn up around the SSO to *E*-AFM phase boundary. Indeed, recent theoretical simulation and experimental observations have revealed some evidences that the multiferroic phase separation indeed occurred around this phase boundary^20^,^26^,^31^, but a systematic study of such a transition and the underline physical mechanism remain uncompleted.

In the present work, orthorhombic Dy_1−x_Ho_x_MnO_3_ (DHMO) (*x* from 0 to 1) polycrystalline samples will be studied to trace the phase transition from the SSO to *E*-AFM phases. Comparing with the extensively studied Eu_1−x_Y_x_MnO_3_ (EYMO) system^20^,^31^,^32^,^33^,^34^,^35^, DHMO is more interesting considering the following factors. First, since the size difference between Dy^3+^ and Ho^3+^ is quite small^21^, the quenching disorder in DHMO is much weaker than that in EYMO, which ensures intrinsic behaviors during the phase transition. In other word, both DMO and HMO locate very close to the phase boundary between the spiral and *E*-AFM^20^,^22^, giving rise to a subtle phase equilibrium in DHMO. Second, both Dy^3+^ and Ho^3+^ are magnetic with large 4*f* magnetic moments while the (Eu_1−x_Y_x_)^3+^ is non-magnetic^32^,^36^,^37^,^38^,^39^,^40^,^41^,^42^,^43^. The strong interaction between the Mn-3*d* spins and Dy/Ho-4*f* spins makes multiplicate origin of ferroelectricity in Dy(Ho)MnO_3_^26^,^27^,^28^,^29^,^30^. So, one can expect that the Mn-Mn exchange interaction together with the *R*-Mn exchange interaction will make the phase transition from the SSO phase to *E*-AFM phase in DHMO with more unique features than that of the EYMO system. These factors allow DHMO a favored candidate to be investigated.

Moreover, we have to clarify that the present work is not a following extension of our previous report on Dy_1−x_Ho_x_MnO_3_ (x ≤ 0.3) although the topic seems to be similar^28^. First, the phase transition and phase separation to be discussed in the present work was not covered in our previous publication^28^. Second, the Dy_1−x_Ho_x_MnO_3_ compound studied in Ref. 28 was synthesized through the traditional solid-state reaction, which restricted the substitution *x* within a low level (*x* ≤ 0.3) due to the unstability of orthorhombic structure of *R*MnO_3_ when *R* is too small. In contrast, the sol-gel sintering technique was adopted in the present work, which can stabilize the orthorhombic structure upon continuous modulation of the ionic radius from Dy to Ho (0 ≤ *x* ≤ 1) even for the meta-stable orthorhombic HoMnO_3_. This process enables us to study the phase transition and phase separation in Dy_1−x_Ho_x_MnO_3_ (0 ≤ *x* ≤ 1) system.

## Results and Discussion

**Figure 1:**
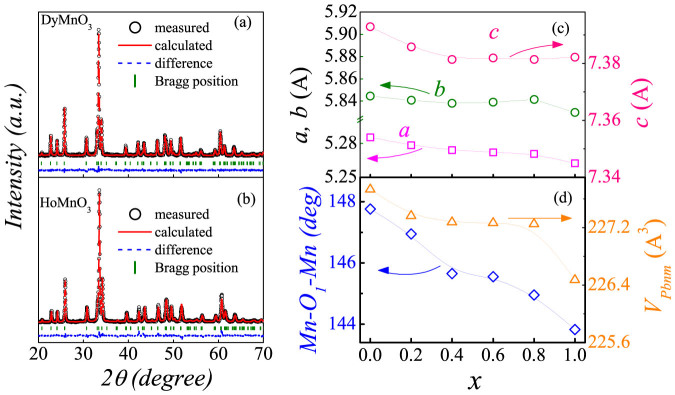
The evaluated structural information of a series of samples Dy_1−x_Ho_x_MnO_3_ (0 ≤ *x* ≤ 1). The XRD patterns and Rietveld refined spectra for (a) DyMnO_3_ and (b) HoMnO_3_ samples, respectively. (c) The obtained lattice parameters of *a, b*, and *c* as a function of *x*. (d) The evaluated lattice volume *V* and Mn-O_1_-Mn bond angle as a function of *x*.

First, in order to trace the evolution of crystal structure of DHMO, the X-ray diffraction patterns obtained at room temperature were refined using the Rietveld analysis. All the X-ray patterns shown here reveal the orthorhombic structure (*Pbnm*) and no impurity phases were detected within the apparatus resolution. Two typical XRD results for samples *x* = 0 and 1 are shown in Figs. 1(a) and 1(b), respectively. The very small difference between the measured spectra and refined ones is insured by the refinement parameter *R_wp_* = 6.05% with lattice parameters *a* = 5.2854Å, *b* = 5.8443Å, and *c* = 7.3927Å for *x* = 0, and *R_wp_* = 8.58% with *a* = 5.2623Å, *b* = 5.8299Å, and *c* = 7.3821Å for *x* = 1. For other samples, the obtained *R_wp_*'s are in the similar level and the obtained lattice parameters of *a*, *b* and *c* are displayed in Fig. 1(c). In general, the unit-cell volume of DHMO sample decreases monotonously (although not linearly) with increasing *x*, as expected and presented in Fig. 1(d)^20^. Furthermore, the Mn-O-Mn bond angles can be also fitted from the structural refinement data, and the evaluated *x*-dependence of the Mn-O_1_-Mn bond angle is also displayed in Fig. 1(d). The successive decrease of this angle is decisive to the development of magnetic ground state from the SSO to *E*-AFM order^35^. This structural evolution is crucial to understand the substitution induced modulation of magnetic order and the associated multiferroicity.

**Figure 2:**
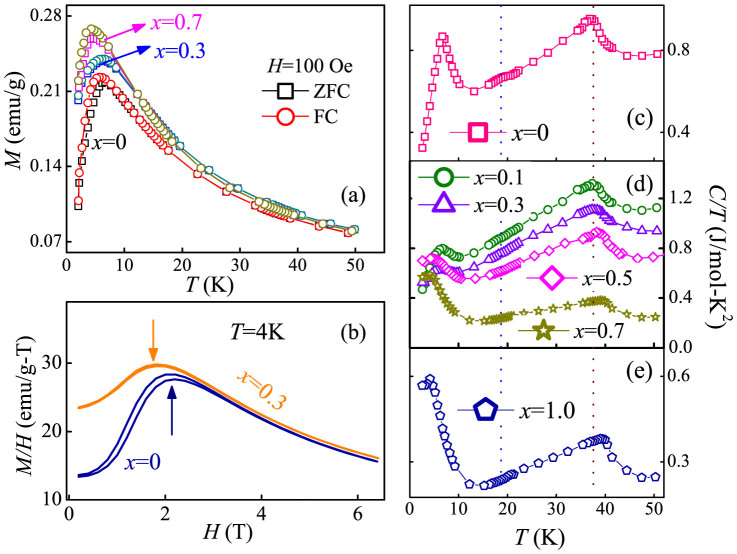
The evolution of the magnetism and heat capacity of Dy_1−x_Ho_x_MnO_3_ (0 ≤ *x* ≤ 1) samples. (a) Measured *M-T* curves under the ZFC and FC conditions for the *x* = 0, 0.3, and 0.7 samples. (b) The *M/H-H* curves for the *x* = 0 and 0.3 samples obtained at *T* = 4 K. (c–e) Measured *C/T-T* plots for (c) *x* = 0, (d) *x* = 0.1–0.7 and (e) *x* = 1, respectively.

Subsequently, we investigate the *x*-dependence of magnetization (*M*) and specific-heat (*C*) as a function of *T*. Here *M* is measured under the zero-field-cooling (ZFC) and field-cooled (FC) conditions with a magnetic field *H* = 100 Oe. As shown in Fig. 2(a), the magnetic behavior of DMO prepared using the sol-gel sintering technique is quite similar to earlier report^27^,^28^,^29^. For example, the pure DMO sample only exhibits a peak at *T = T_Dy_* ~ 6.5 K in both the ZFC and FC cycles due to the dominating Dy^3+^ spin moment. The measured *M-T* curves for the *x* = 0.3 and 0.7 samples show that the antiferromagnetic (AFM) transition occurring at *T_Dy_* downshifts with increasing *x*, implying the suppression of the independent Dy^3+^ spin order. Since Ho^3+^ carries a magnetic moment slightly larger than that of Dy^3+^ 38,39,44, the enhancement of measured *M* signals upon Ho substitution also confirms the dominating role of Dy^3+^/Ho^3+^ spin moment in deciding the magnetization of DHMO. Furthermore, the isothermal magnetization curves measured at *T* = 4 K for the *x* = 0 and 0.3 samples are shown in Fig. 2(b). The curve of *x* = 0 indicates a metamagnetic transition around 2.2 T. This metamagnetic transition corresponds to the change of stacking mode of Dy^3+^ moments, which may be responsible for the modulation of *P* in DMO upon magnetic field^38^,^39^. As for the magnetization of *x* = 0.3, this metamagnetic transition becomes faint and downshifts to around 1.8 T, indicating the suppression of the independent Dy^3+^'s magnetic ordering upon Ho's substitution.

The temperature dependence of heat capacity, plotted as *C*/*T*, is depicted in Figs. 2(c)–2(e), for the *x* = 0, 0.1–0.7 and 1.0 samples, respectively. For the pure DMO (*x* = 0), an anomaly is observed at *T_N_* ~ 37 K, which corresponds to the phase transition of Mn spins from paramagnetic (PM) state into incommensurate sinusoidal collinear antiferromagnetic (IC-AFM) spin order phase^9^,^22^. A minor second anomaly at *T = T_FE_* ~ 18 K is the signature of the Mn's cycloidal spin order plus the affiliated Dy's cycloidal order with a temperature-independent wavevector *τ*^Mn^ = 0.385 (according to previous neutron studies), below which a finite *P* emerges^42^. Upon further cooling down to *T = T_Dy_* ~ 6.5 K, one more major anomaly of *C/T* associated with the independent Dy's spin ordering is identified^9^, in consistent with the peak position in the *M-T* curve. These successive phase transitions are in agreement with earlier reports^9^,^38^,^39^,^40^,^41^,^42^,^43^. Regarding the substituted compounds, the anomaly at *T_N_* shifts to higher temperatures since HMO has its *T_N_* ~ 41 K which is higher than that of DMO^44^. However, with increasing *x*, the onset point of ferroelectricity (*T_FE_*) cannot be detected above *x* = 0.3, clearly marking the severe suppression of the cycloidal spin order upon the higher Ho substitution. It is also indentified that *T_Dy_* gradually downshifts to lower *T* range, which provides a clear evidence for the suppression of the long range order of *A*-site Dy^3+^/Ho^3+^ spins induced by the Ho substitution.

**Figure 3:**
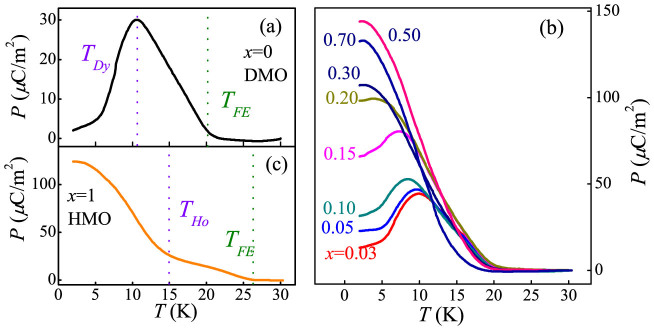
The temperature-dependent ferroelectric polarizations of the Dy_1−x_Ho_x_MnO_3_ (0 ≤ *x* ≤ 1) samples: (a) *x* = 0, (b) 0.03 ≤ *x* ≤ 0.7 and (c) *x* = 1, respectively.

We then pay attention to the ferroelectricity. Figs. 3(a)–(c) show the *T*-dependence of *P* for various DHMO samples, measured via the pyroelectric method after the *E* = 10 kV/cm field poling. For pure DMO (Fig. 3(a)), *P* appears around *T_FE_* ~ 18 K due to the spatial symmetry breaking induced by the spiral spins orders of Mn and Dy^42^. After reaching the maximum value (~30 μC/m^2^), the *P* is severely suppressed below 10 K, due to the emergence of independent collinear AFM order of Dy^3+^'s spins. The decoupled Dy-Mn spin pairs result in the decreased *P* below *T_Dy_*^27^,^28^,^29^,^38^,^39^,^40^,^41^,^42^,^43^. Here, the measured *P* of DMO is smaller than its bulk counterpart in the whole temperature region^28^, which can be attributed to more grain boundaries and smaller grain size due to the low crystallization temperature used in the sol-gel sintering method. As demonstrated in previous literature, when the grain size is reduced, the increased surface disorder and defects at grains boundaries will not only prevent the formation of the long-range FE order but also can lead to the clamping of domain walls^45^,^46^,^47^. Thus, the small measured *P* value in our present experiment is physical reasonable. Furthermore, the behavior of *P* in our experiment is very close to those single crystalline one^30^, offering a reliable platform to further investigate the effects of *R*-Mn spin coupling and the possible phase separation in these multiferroic manganites.

The measured *P-T* curves of DHMO (0.03 ≤ *x* ≤ 0.7) samples are displayed in Fig. 3(b) Interestingly, the prominent kink in DMO, gradually fades away in the 0.03 ≤ *x* ≤ 0.15 region and cannot be detected anymore when *x* ≥ 0.2. Thus, the evolution of such a kink indicates the suppression of the independent Dy^3+^ spin ordering upon the Ho substitution, in consistent with above magnetic and specific-heat measurements. As a comparative one, the *P-T* curve of HMO (*x* = 1) is displayed in Fig. 3(c). Clearly, the *P* of HMO emerges since *T_FE_* = 27 K, the temperature of the lock-in transition of Mn spins into the *E*-AFM phase^36^,^37^, and climbs rapidly only below *T_Ho_* = 15 K, the temperature at which the magnetic structure of Ho^3+^ spins is formed. It should be noted that there are debates on the actual spin structure of orthorhombic HoMnO_3_. A new spin structure with wave vector of *k* = 0.4 has been reported in orthorhombic HoMnO_3_^30^, giving a *FE* polarization induced by the Mn-Ho exchange striction only below 15 K. Considering the fact that the *FE* polarization appears since 27 K in our experiments, our orthorhombic HoMnO_3_ sample should be with the *E*-AFM order^36^,^37^. In HMO, *P* is originated from the Mn-Mn symmetric exchange striction, but the significant increase of *P* is contributed to the Ho-Mn exchange striction^30^,^36^,^37^, implying that the *R*-Mn spin coupling is also a crucial role in deciding the ferroelectric properties of *R*MnO_3_, not only in DMO but also in HMO. In general, the measured *P-T* curves of DHMO (0 ≤ *x* ≤ 1) compounds show that the initial SSO transforms into the final *E*-AFM structure. Since the exchange striction is much stronger than the Dzyaloshinskii-Moriya interaction^18^,^19^, the magnetic evolution from the SSO to *E*-AFM is expected to enhance the *P* of DHMO samples.

**Figure 4:**
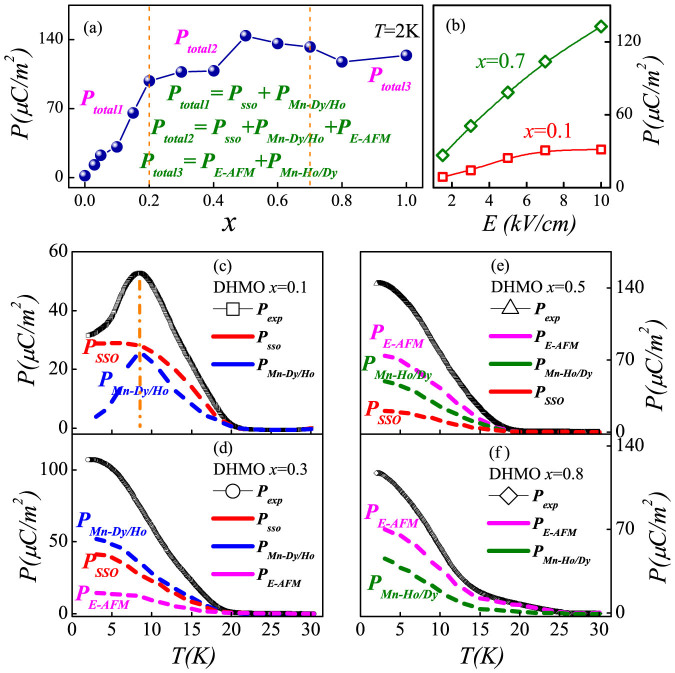
(a) The measured *P(x)* plot of Dy_1−x_Ho_x_MnO_3_ samples with 0 ≤ *x* ≤ 1 at *T* = 2 K, which can be roughly partitioned into three regions according to the origin of total *P*. (b) *P(E)* curves for *x* = 0.1 and 0.7 obtained at *T* = 2 K. (c–f) Sketch maps of the temperature-dependence of various polarization components of DHMO samples with *x* = 0.1, 0.3, 0.5 and 0.8. *P_sso_* represents the contribution from the spiral spin order of Mn spins, *P_E-AFM_* denotes the contribution from the *E*-type AFM of Mn spins, *P_Mn-Dy/Ho_* is the contribution from the exchange striction between the Mn-Dy or Mn-Ho spin pairs. *P_total_* is the total polarization from measurement. The partition is quantitive reference only.

To clearly illustrate the enhancement of polarization, the *x*-dependence of *P* obtained at *T* = 2 K (below *T*_Dy/Ho_) is presented in Fig. 4(a). With increasing *x*, the measured *P* increases rapidly in the low Ho region (*x* ≤ 0.2) but slowly in higher Ho region (0.2 < *x* ≤ 0.4). Interestingly, with further increasing Ho substitution, an obvious climbing of *P* is identified between *x* = 0.4 and *x* = 0.5. However, after the maximum *P* which is significantly enhanced up to 144 μC/m^2^ at *x* = 0.5, a slight decline of *P* is observed for higher concentration *x* > 0.5, which could be partially attributed to the different saturated fields required to pole the samples. As shown in Fig. 4(b), the *P-E* dependence is different between the *x* = 0.1 and 0.7 samples: the *P* for *x* = 0.1 trends to saturate under *E* = 10 kV/cm while the saturated poling field needed for *x* = 0.7 should be higher than 10 kV/cm. Therefore, the measured *P* for those *x* > 0.5 samples may be not fully saturated, giving rise to the slight decline of *P*.

In the following, the evolutions of ferroelectric sources are qualitatively analyzed from the viewpoint of phase transition and phase separation between the SSO and *E*-AFM. As stated before, the origin of ferroelectric *P* in DHMO can be multifold, varying as a function of temperature and substitution. Intuitively, a sketch map of the *T*-dependence of various polarization components at *x* = 0.1 is qualitatively displayed in Fig. 4(c). In short, one can infer that within the low substitution range, e.g. 0 < *x* ≤ 0.2, the ferroelectrity of DHMO sample is mainly from the SSO induced one plus the exchange striction one between Mn and *R*: *P_total1_* = *P_sso_* + *P_Mn-Dy/Ho_*, both of which are enhanced by Ho's substitution.

With further increase of Ho's substitution (0.2 < *x* ≤ 0.4), the difference in ionic radius between Dy and Ho leads to suppression of the original SSO. Accompanying the gradually weakened SSO of Mn spins, the strength of *J_Mn-Ho_* becomes stronger. Moreover, a faint *E*-AFM spin structure may gradually emerge in this substitution region and contributes a little to the total *P* of DHMO samples. These factors may be responsible for the slow growth of *P* in this substitution range. A sketch map of the selected *x* = 0.3 sample is displayed in Fig. 4(d). Qualitatively, the total *P* of DHMO samples (0.2 < *x* ≤ 0.4) can be expressed as *P_total2_* = *P_sso_* + *P_Mn-Dy/Ho_* + *P_E-AFM_*. Under heavy Ho substitution (0.4 < *x* < 0.8), the SSO of Mn spins collapses and disappears gradually, and the spin structure transforms into the *E*-type AFM mostly. The sketch map of the *T*-dependence of various polarization components of the special *x* = 0.5 sample is shown in Fig. 4(e). Following this sketch, the *P* of DHMO samples with Ho substitution (0.4 < *x* < 0.8) range can be also expressed as *P_total2_* = *P_sso_* + *P_Mn-Dy/Ho_* + *P_E-AFM_*, although the weights of these three items have changed. Then, let us pay a little more attention to the obvious climbing of *P* observed within 0.4 < *x* < 0.5, as shown in Fig. 4(a). This phenomenon seems to be a signal of prominent phase separation between the SSO and *E*-type AFM. The SSO is rapidly replaced by the *E*-AFM one with increasing *x* in this region, which will result in a significant enhancement of total *P* and sensitive magnetoelectric response (to be studied below). With further increase of Ho's substitution (0.8 ≤ *x* ≤ 1), the *E*-AFM of Mn spins becomes completely stabilized while the SSO of Mn spins completely disappears in these DHMO samples. As shown in the sketch map for the *x* = 0.8 sample (Fig. 4(f)), the formulation of *P* can be written as: *P_total3_* = *P_E_*_-AFM_ + *P_Mn-Ho/Dy_*. Of course, the partition of total *P* shown in Fig. 4 is not quantitatively rigorous, but for qualitive reference only. More systematic and precise studies need direct measurements (e.g. using neutron scattering) of spin orders, which are beyond the current work. Even though, our work can still provide a simplified physical scenario to describe the phase transition and phase separation in multiferroic *R*MnO_3_.

**Figure 5:**
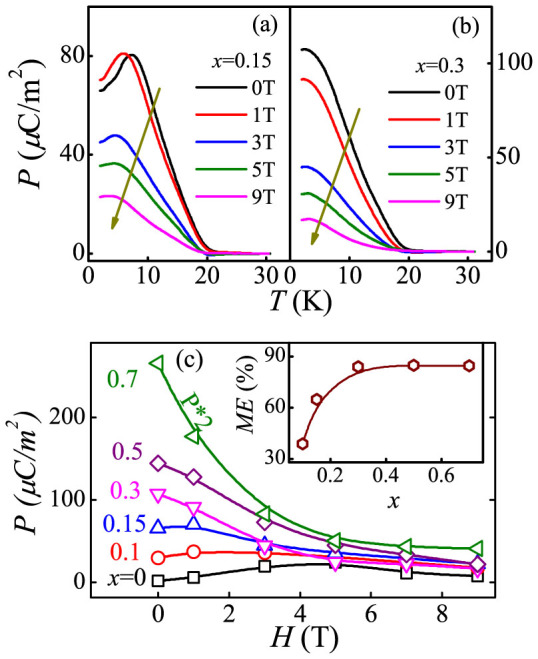
Ferroelectric *P* response to magnetic field for selected Dy_1−x_Ho_x_MnO_3_ samples. Measured *P(T)* curves for (a) *x* = 0.15 and (b) *x* = 0.3 under magnetic fields. (c) *P(H)* for selected samples at *T* = 2 K. Inset: the ME coefficient as a function of *x* obtained at *T* = 2 K and *H* = 9 T.

To further check the physical behavior accompanying the phase transition and phase separation, the response of *P* to external magnetic field (*H*) for DHMO samples are measured in detail. The *P-T* curves measured under different magnetic fields for selected samples *x* = 0.15 and 0.3 are shown in Figs. 5(a) and 5(b). For the *x* = 0.15 sample, the measured *P* at low temperatures is enhanced firstly and then suppressed when the magnetic field is higher than 1 T. This enhancement under weak magnetic fields is a fingerprint of DMO, due to the suppression of Dy^3+^'s independent spin order. In contrast, the magnetic field response of *P* for the *x* = 0.3 sample is nontrivially different, which is dramatically reduced under external magnetic field. Furthermore, the *H*-dependence of *P* at *T* = 2 K for selected compounds is investigated. As indicated in Fig. 5(c), *P* evolves with *H* in a similar way for the *x* ≤ 0.15 samples while it exhibits a different evolution for the *x* ≥ 0.3 samples, implying the different source of ferroelectric *P*.

**Figure 6:**
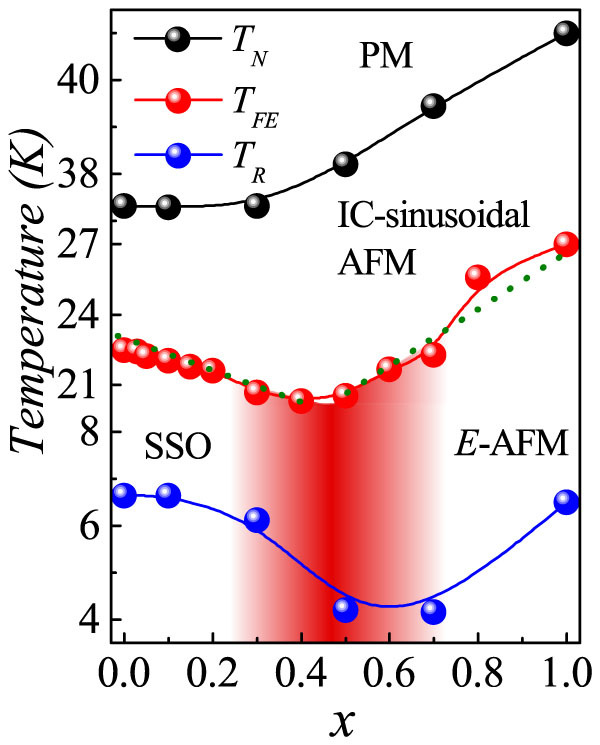
The multiferroic phase diagram of Dy_1−x_Ho_x_MnO_3_. *T_N_* and *T_Dy/Ho_* derive from the *C/T* measurement, while *T_FE_* is obtained from the ferroelectric measurement. PM, IC-AFM, SSO, and *E*-AFM denote the paramagnetic, incommensurate-antiferromagnetic, spiral and *E*-type antiferromagnetic spin orders, respectively. The shadow region indicates the possible phase separation area.

Based on above magnetic and ferroelectric measurements as well as the heat capacity, we are allowed to establish a sketch of the multiferroic phase diagram in the temperature-substitution (*T*-*x*) space, as shown in Fig. 6. Here, one note that the *T_N_* increases monotonously with *x*, implying the PM to IC-AFM transition is uniform for all DHMO samples. The most interesting feature is the ferroelectric transition temperature *T_FE_*, below which the system becomes a multiferroic, displays a V-shaped evolution along with *x*: downshifts firstly as *x* increases from 0 to 0.4 and then tends to increase above 0.5, suggesting the substitution-induced first-order phase transition. Under appropriate Ho substitution levels, e.g. 0.4 < *x* < 0.5, the phase separation between SSO and *E*-AFM orders should be prominent in DHMO, as the reason for the aforementioned abnormal magnetism/ferroelectric behaviors^20^. And the trace of such a phase separation between SSO and *E*-AFM may survive even within a wider region, as analysized above and sketched in Fig. 6. For example, the tiny upturn of *T_FE_* from *x* = 0.7 to *x* = 0.8 may be a signal of the complete disappear of SSO.

According to this phase diagram, we are allowed to clarify the origin of the *P* enhancement and the significant modulation of the response of *P* against *H* upon Ho substitution. Keeping in mind the Rietveld refinement results shown in Fig. 1, the volume of the unit cell shrinks upon the substitution of Dy by Ho but with a terrace around 0.4 < *x* < 0.8, coinciding with the possible phase coexistent between the SSO and *E*-AFM phases. However, the Mn-O_1_-Mn bond angle continuously decreases upon the Ho substitution, suggesting an increase of the buckling and tilting angles of the MnO_6_ octahedra^22^,^35^. The enhanced distortion of the orthorhombic structure will lead to the magnetic transition from the *bc*-cycloidal to the *E*-AFM phase through the possible phase coexistence states^20^,^22^.

Under small Ho concentrations *x* ≤ 0.2, a sharp shrinking of the unit cell volume as well as the decreasing of the Mn-O_1_-Mn bond angles is observed, allowing for a further frustration of the spin structure, which benefits to the enhancement of *P* by shortening the spin spiral period^20^,^22^. Furthermore, the suppression of the independent Dy^3+^'s spin order is also beneficial to the *P*-enhancement at low temperatures^27^,^28^,^29^,^38^,^39^. At this stage, the dominated ground state of the *bc*-cycloidal phase is preserved, evidenced by the slight enhancement of *P* observed under intermediate *H* at low *T*, as seen in Fig. 5(a). However, for higher concentration, *x* = 0.3, the local SSO structure is gradually destroyed since the onset point of the ferroelectric polarization *T_FE_* becomes faint and cannot be detected in our heat-capacity measurements [Fig. 2(d)]. The applied external magnetic field accelerates the collapse of the original SSO structure. These are the reasons for the *P* suppression against increased *H* in *x* = 0.3 compound [Fig. 5(b)]. Further increasing Ho substitution to *x* = 0.5, the spin structure of DHMO will transform into *E*-AFM phase mostly, identified in Fig. 2(d) that the *C/T-T* curve of *x* = 0.5 exhibits similar evolution to that of HMO. Since the *P* of the *E*-AFM HMO is demonstrated to be larger than that of the *bc*-cycloidal phase^20^, obvious enhancement of *P* is reasonably expected in DHMO samples with *x* ≥ 0.5. Moreover, in the *E*-AFM HMO, the spin structure of Ho^3+^ can be rearranged under external magnetic field^36^,^37^. So, a strong magnetic field response of the polarization can be expected in DHMO samples with *x* ≥ 0.5. As shown in Fig. 5(c), the ME coefficient, defined as (*P*(0) − *P*(*H*))/*P*(0), is found to be dramatically enhanced up to ~84% at *H* = 9 T and *T* = 2 K for *x* = 0.3 and trends to saturate as *x* > 0.3.

In conclusion, we have performed detailed experimental investigations on the magnetism, specific heat, and electric polarization of the multiferroic Dy_1−x_Ho_x_MnO_3_. Through systematically tuning the *A*-site ionic radius and magnetism, the substitution of Dy^3+^ with Ho^3+^ ions leads to the continuous phase transition of the ground state from the cycloidal to the *E*-AFM phase, as well as the enhancement of ferroelectric polarization and magnetoelectrc response, distinguishing the DHMO system from the others. Based on these measurements, a phase diagram for Dy_1−x_Ho_x_MnO_3_ compositions have been constructed in the whole range 0 ≤ *x* ≤ 1. According to the macroscopic behaviors, the phase separation effect is expected to be prominent within 0.4 < *x* < 0.5, while its trace can survive to a wider region. This system offers the possibility to complete the multiferroic phase diagram and tune the multiple ferroelectricity of *R*MnO_3_.

## Method

Orthorhombic DHMO samples in the whole 0 ≤ *x* ≤ 1 region were prepared by the sol-gel sintering technique. It should be noted that *R*MnO_3_ with a smaller *R* cation than Dy^3+^ (e.g. Ho^3+^) usually crystallizes into a hexagonal structure^48^. Usually a high pressure technique is needed in the conventional solid state reaction to obtain a pure orthorhombic phase of HMO. Alternatively, the sol-gel sintering approach provides as a feasible way to obtain the pure orthorhombic phase. In the sol-gel process, the crystallization temperatures above 1000°C were not used to avoid the formation of hexagonal HMO^49^, thus the final crystallization temperature was set to 850°C. Under this synthesis condition, the as-prepared DHMO samples were well-crystallized to the orthorhombic structure. Also due to such a relative low crystallization temperature, the grain sizes are about 150 nm, smaller than that of bulk samples synthesized by solid state reaction. X-ray diffraction (XRD) with Cu *Kα* radiation was performed to confirm the phase purity. The magnetic and specific-heat measurements were conducted employing the Superconducting Quantum Interference Device (SQUID) and Physical Properties Measurement System (PPMS), respectively. To measure *P*, the temperature-dependence of pyroelectric current was detected using Keithley 6514 electrometer in PPMS. The poling electric field is *E* = 10 kV/cm and more details of the measuring procedure were reported earlier^50^.

## Author Contributions

N.Z., S.D. and J.M.L. conceived and designed the experiments. N.Z. and Z.B.Y. carried out the experiments. N.Z., S.D. and J.M.L. wrote the paper. Z.M.F. and F.G.C. reviewed and commented on the paper. All authors discussed the results and commented on the manuscript.
